# Validity of registration of ICD codes and prescriptions in a research database in Swedish primary care:  a cross-sectional study in Skaraborg primary care database

**DOI:** 10.1186/1472-6947-10-23

**Published:** 2010-04-23

**Authors:** Per Hjerpe, Juan Merlo, Henrik Ohlsson, Kristina Bengtsson Boström, Ulf Lindblad

**Affiliations:** 1 R&D Centre, Skaraborg Primary Care, Skövde, Sweden; 2 Social Epidemiology, Department of Clinical Sciences, Faculty of Medicine, Lund University, Sweden; 3 Unit for Primary Health Research, Region Skåne, Sweden; 4 Department of Public Health and Community Medicine/Primary Health Care, The Sahlgrenska Academy at the University of Gothenburg, Sweden

## Abstract

**Background:**

In recent years, several primary care databases recording information from computerized medical records have been established and used for quality assessment of medical care and research. However, to be useful for research purposes, the data generated routinely from every day practice require registration of high quality. In this study we aimed to investigate (i) the frequency and validity of ICD code and drug prescription registration in the new Skaraborg primary care database (SPCD) and (ii) to investigate the sources of variation in this registration.

**Methods:**

SPCD contains anonymous electronic medical records (ProfDoc III) automatically retrieved from all 24 public health care centres (HCC) in Skaraborg, Sweden. The frequencies of ICD code registration for the selected diagnoses diabetes mellitus, hypertension and chronic cardiovascular disease and the relevant drug prescriptions in the time period between May 2002 and October 2003 were analysed. The validity of data registration in the SPCD was assessed in a random sample of 50 medical records from each HCC (n = 1200 records) using the medical record text as gold standard. The variance of ICD code registration was studied with multi-level logistic regression analysis and expressed as median odds ratio (MOR).

**Results:**

For diabetes mellitus and hypertension ICD codes were registered in 80-90% of cases, while for congestive heart failure and ischemic heart disease ICD codes were registered more seldom (60-70%). Drug prescription registration was overall high (88%). A correlation between the frequency of ICD coded visits and the sensitivity of the ICD code registration was found for hypertension and congestive heart failure but not for diabetes or ischemic heart disease.

The frequency of ICD code registration varied from 42 to 90% between HCCs, and the greatest variation was found at the physician level (MOR_PHYSICIAN _= 4.2 and MOR_HCC _= 2.3).

**Conclusions:**

Since the frequency of ICD code registration varies between different diagnoses, each diagnosis must be separately validated. Improved frequency and quality of ICD code registration might be achieved by interventions directed towards the physicians where the greatest amount of variation was found.

## Background

Quality assessment is fundamental for maintaining an effective health care system and is therefore a major focus of attention in many health care systems. An increasing number of databases that record information from computerized medical records from health care centres (HCCs) are being established in many countries [1-4]. These databases include information such as clinical diagnoses, laboratory analyses and medical treatments including prescribed medication. However, to be useful for research purposes or auditing of health care, the registration must be of high quality, which may be difficult to attain when the information is routinely generated in every day practice.

The Skaraborg Primary Care Database (SPCD) was initiated in the year 2000 by linking information from the 24 public health care centres (HCCs) in the county of Skaraborg in Sweden. SPCD was one of the first large databases of this kind launched in Sweden.

In this database, diagnoses are coded according the Swedish version of the 10^th ^version of the International Classification of Diseases (ICD-10) adapted for primary care [5]. The frequency of visits with a coded diagnosis is an established measure of quality. A previous study has shown that the frequency of ICD codification varies between HCCs and between diagnoses [6]. For example, two different HCCs could have the same overall frequency of ICD coding but very different frequencies of coding for different diagnoses. Further, we have found no study focusing on the role that different health care levels (e.g., patient, physician, HCC) play for understanding differences in ICD coding at the visit level.

On this background, we set out to assess the frequency and sensitivity of visit ICD coding and recorded prescriptions in the SPCD for four different diagnoses; hypertension, diabetes mellitus, congestive heart failure (CHF) and ischemic heart disease (IHD). Furthermore, we performed a multilevel logistic regression analysis to quantify the relative importance of different levels (patient, physician, HCC) for understanding variations in ICD coding.

## Methods

### Study population and the Skaraborg Primary Care Database (SPCD)

The county of Skaraborg is situated in the region of Västra Götaland in the southwest of Sweden, and has a population of approximately 250000 inhabitants. The county is mostly rural and is divided in 15 municipalities. Primary care is supplied by one private and 24 public HCCs, as well as by a few private GPs. About 250000 office visits are registered in the public HCCs every year. In 2007, 75% of all drug prescriptions were issued by the primary health care, and 85% of these prescriptions were made at the public HCCs.

Since year 2000, all 24 public HCCs in Skaraborg primary care share the same computerized medical record system, Profdoc Journal III 1.82 (Profdoc AB, Uppsala, Sweden, PDIII). Primarily, this computerized medical record was intended for clinical purposes and therefore all HCCs have a separate electronic record database with local accessibility. The free text part of the patient record, which includes all visit notes, is normally written by the secretary from the physician's dictation. Hospital letters are scanned into the patient's record so no information is stored in paper form. Laboratory results are recorded partly automatically and partly manually by the laboratory staff.

The ICD codes are assigned by the physician at the time of the visit and should reflect all health problems addressed during the visit. The ICD codes are registered by the physician during the patient's visits or later by the secretary from the physician's dictation. The ICD codes are selected from a list included in the PDIII medical record software. While the coding of all patient visits is considered routine in order to enhance quality assessment, there are no incentives for ICD coding in primary care. According to Swedish law [7], medical records must include coding of all health care contacts in hospital care but not in Primary Care. Furthermore, in 2003 there were no economical or other incentives for coding since primary care reimbursement was totally independent of coding performance. Prescription information is automatically recorded in the medical records at the time of prescription and includes the name of drug, its Anatomical Therapeutical Chemical (ATC) code, and the amount of drug prescribed. Medication prescribed to patients cared for in municipal home care outside of the HCCs is not recorded in PDIII. For cardiovascular drugs, the proportion of drugs prescribed by the municipal homecare system, and therefore not included in the PDIII database, varies by age, being approximately 5% in patients less than 80 years of age and about 35% in patients aged 80 years and more.

**Figure 1:**
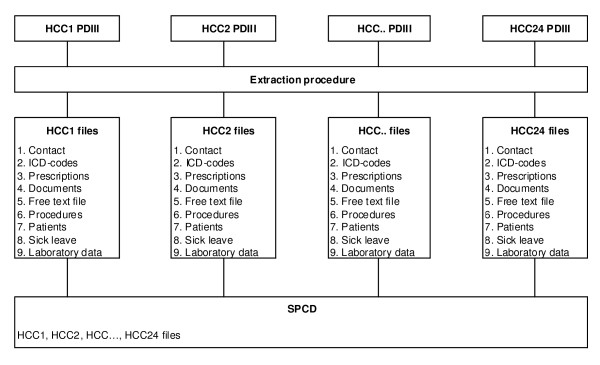
**The compilation procedure of Skaraborg Primary Care Database (SPCD)**. PD III = ProfDoc Journal III 1.82, HCC = Health Care Centre.

In the SPCD all medical record information is regularly extracted from the local PDIII databases in the 24 HCCs by a purpose-built software (Figure 1). The retrieval of data from the local PDIII databases to the SPCD is done automatically without direct involvement of the individual physician. Patient and staff identities are blinded and are assigned specific dummy identification numbers to allow the linkage of the information within the database. During the extraction procedure, nine separate files containing laboratory data, drug prescriptions, ICD codes, contact information, documents (referral letters), part of the free text (e.g. blood pressure), therapeutic procedures, information on sick leave and postal codes are retrieved from each of the 24 local PDIII databases.

### Statistical and epidemiological analyses

#### Validation of ICD codes and prescriptions

We selected all patients in the SCPD with at least one prescription for cardiovascular drugs (Table 1), from 1^st ^May 2002 to 31^st ^October 2003. In these patients we identified all the ICD-10 codes for diabetes mellitus (E118P, E119, E108P, E14-P, E109), hypertension (I10-, I13-P), ischemic heart disease (I25-P, I209P, I21-P, I200-) and congestive heart failure (I50-). A random sample of 50 patients from each HCC was drawn from the selected patients and the information on diagnoses and prescribed drugs in the free text part of the electronic PDIII journal was used as gold standard for assessing the validity of the ICD codes and prescribed drugs found in the SPCD. The free text includes all notes from visits, telephone contacts, and any other situation of relevance for the care of the patient. The free text part of the electronic journal also includes an automatically written text that is generated when diagnoses codes or medications are registered. Therefore, all diagnoses and medications registered in the designated code field of the electronic journal are automatically recorded as free text as well. On the contrary, diagnoses or medications noted only in the free text section of the journal do not generate an ICD code. Therefore, since the database is constructed with information from the specific code fields of the electronic medical records, any diagnosis that only appears in the free text part of the journal will be missed.

**Table 1:** Drug groups included in the study.

Drug groups	ATC codes
Long-acting nitrates	C01DA08, C01DA14
Loop diuretics	C03C
Potassium-sparing diuretics	C03D
Diuretic combinations	C03E
Thiazides	C03A, C03B
Beta blockers	C07
Calcium channel blockers	C08
ACE-inhibitors	C09A, C09B
Angiotensin receptor blockers	C09C, C09D
Statins	C10AA
Fibrates	C10AB
Resins	C10AC

Drug groups with ATC codes (Anatomic Therapeutic Chemical classification system).

To evaluate the validity of the SPCD we compared the information in the files extracted into the SPCD with the information in the free text sections of the electronic medical records. All text from the computerized patient records were transferred from the SPCD database to a spreadsheet (Microsoft Excel) and in a first step a macro was used to highlight relevant words or text fragments (e.g. diabetes, metoprolol) to facilitate the second step where the complete texts were visually reviewed to identify relevant diagnoses and prescriptions.

**Figure 2:**
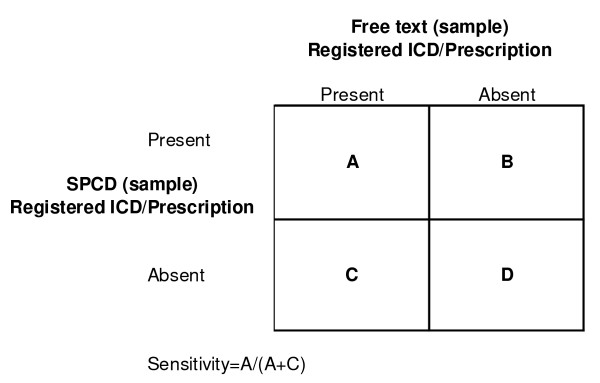
**Relationship between Gold standard (Journaltext in PDIII) and Test outcome (ICD codification and recorded prescriptions in SPCD)**.

*The sensitivity of the ICD coding *in the SPCD was calculated as the percentage of patients with relevant diagnoses or prescriptions in the free text section of the medical records that had a matching ICD or ATC code in the SPCD (Figure 2).

A = patients with specific ICD codes/prescriptions in the SPCD

A+C = patients with specific diseases/prescription in the free text

Sensitivity = A/(A+C) * 100

*The frequency of visits with ICD codification *in the SPCD was computed by dividing the number of visits with a registered ICD code by the total number of visits in each HCC during the study period (1^st ^May 2002 to 31^st ^March 2003).

T = total number of visits

N = number of coded visits

N/T = frequency of ICD coded visits

To determine the strength of linear dependency between frequency of ICD coded visits and the sensitivity of ICD coding and registration of medication we calculated Pearson correlation coefficient (r).

#### Multilevel logistic regression analysis

We extracted information on ICD coding performed by the 858 physicians (approximately 130 employed General practitioners and the rest Interns, Residents or Locums) at the 24 HCCs at all patient visits (n = 348,776) during the study period (1^st ^May 2002 to 31^st ^October 2003) and performed a multilevel logistic regression analysis that accounted for the hierarchial structure of the data with patient visits nested within physicians that in turn were nested within HCCs. These analyses allowed us to observe how variance was partitioned between visits, physicians and HCCs and thereby quantify the relative importance of these levels for variation in ICD coding.

In these analyses, the outcome was a dichotomous variable indicating if a visit had an ICD coding or not. The patients' sex (with women as reference) and age (categorized by quartiles with the youngest age group as reference) were identified as independent variables at visit level. Type of visit was defined as planned or unplanned with planned visit as reference.

We developed two consecutive models. In the first model (A) we only included physicians and HCCs as random effects. In the second model (B) we added the characteristics of the visit as fixed effects, which allowed us to investigate whether these characteristics explained residual variation at the physicians and HCCs levels. In order to quantify the importance of the different levels for ICD coding, we calculated the median odds ratio (MOR) [8,9]. The MOR translates the higher level variance into the widely used odds ratio (OR) scale which has a consistent and intuitive interpretation. In simple terms, the MOR could be interpreted as how much a patient's odds of having an ICD coded visit will (in median) increase if this patient was treated by a physician/HCC with higher inclination for coding of visits. A MOR equal to 1 indicates that there are no differences between physicians/HCCs in their propensity to enter the ICD code at the time of the patient's visit.

To study associations in the fixed effects part of the multilevel logistic regression we calculated ORs and their 95% credible intervals (95% CIs) obtained from the posterior distribution of the regression coefficients. We calculated the percentage of change in the variance (PCV). That is, the percentage of the variance in the initial model (Var_model A_) that was explained when including more variables in an extended model (Var_model B_) as:

Parameters were estimated using Markov chain Monte Carlo (MCMC) methods in the MLwiN software [10] and the Deviance Information Criterion (DIC) was used to evaluate goodness of fit [11].

The study was approved by The Regional Ethical Review Board in Gothenburg.

## Results

### Validation of ICD codes and prescriptions

In the SPCD we identified 32 846 individual patients with prescriptions of drugs for cardiovascular diseases during the study period (Table 2). Of these patients, 58% (18928/32 846) had hypertension, 19% (6082/32 846) presented ischemic heart disease (IHD), 8.2% (2 687/32846) congestive heart failure (CHF), and 16% (5373/32846) diabetes in the SPCD diagnosis register. In order to get a rough estimate of the completeness of ICD coding we used the information from the SPCD and found a prevalence in the population (n = 250000) of 7.6% (18928/250000) for hypertension, 2.4% (6082/250000) for IHD and 1.1% (2687/250000) for CHF. The prevalence of diabetes could not be estimated as only patients with cardiovascular drugs were included in the study, excluding patients with diabetes but no cardiovascular medication.

**Table 2:** Characteristics of included patients and their corresponding ICD codes.

	All patients (n = 32846)	Random sample (n = 1200)	
**Characteristics of patients**			
*Age*			
Range	4-106	28-95	
Median	70	69	
*Sex*			
Female	18206 (55%)	630 (52%)	
Male	14639 (45%)	570 (48%)	
			
**ICD-codes**		*In SPCD*	*After validation*
Hypertension	18928 (58%)	696 (58%)	838 (70%)
Ischemic heart disease	6082 (19%)	245 (20%)	320 (27%)
Congestive heart failure	2687 (8%)	100 (8%)	152 (13%)
Diabetes	5373 (16%)	200 (17%)	225 (19%)
None of these diagnoses	8197 (25%)	283 (24%)	134 (11%)

Extracted from SPCD during 1^st ^May 2002 - 31^st ^October 2003 (n = 32846)

**Figure 3:**
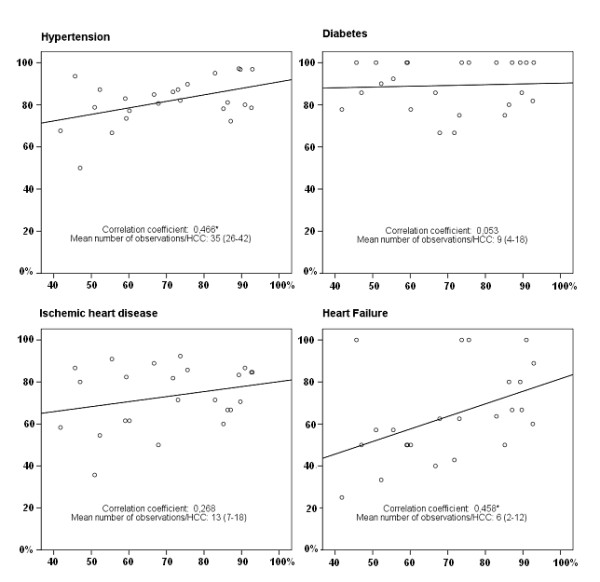
**Correlations between coded visits and patients with correct registration for the different diagnoses**. Correlation between frequencies of ICD coded visits during 2002-2003 and proportion of patients with correct registration for different diagnoses for each of the 24 health care centres in Skaraborg. * Significant at < 0.05 level. On the X axis: Proportion of coded visits. On the Y axis: Proportion of patients with correct registration.

**Figure 4:**
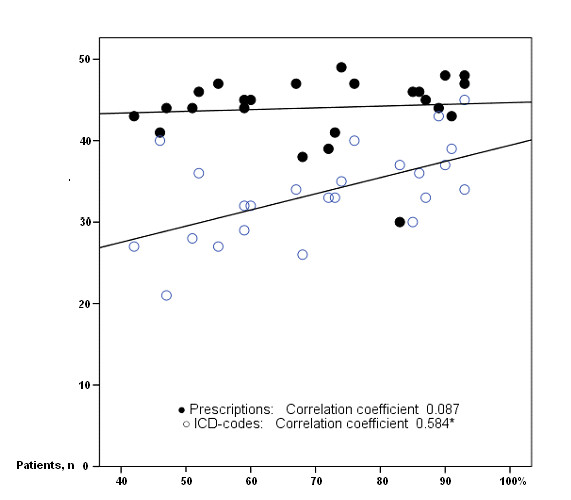
**Correlation between coded visits and patients with correct diagnose and medication registration respectively**. Correlation between frequencies of ICD coded visits during 2002-2003 and number of patients with correct registration of ICD code and medication for the 24 health care centres in Skaraborg. * Significant at < 0.01 level. On the X axis: Proportion of coded visits. On the Y axis: Number of patients with a correct combination of Prescription/ICD code registration respectively.

The random sample of 1200 patient records (50 from each of the HCCs) showed that sensitivity of ICD codes in the SPCD varied between HCCs. For diabetes the sensitivity varied between 67 and 100% (mean 89% (95% CI: 85-93)), for hypertension between 50 and 97% (mean 83% (95% CI: 80-86)), for IHD between 36 and 92% (mean 77% (95% CI: 72-81)), and for CHF between 25 and 100% (mean 66% (95% CI: 58-73)). A correlation between the frequency of ICD coded visits and sensitivity of the ICD files in the SPCD was found for hypertension (r = 0.466) and CHF (r = 0.458) but not for diabetes or IHD (Figure 3). A correlation was also found between the number of patients with a completely correct ICD code combination and the frequency of coded visits (r = 0.584) (Figure 4). The variation of sensitivity in medication registration between HCCs was 60-98% (mean 88% (95% CI: 86-90)), data not shown. There was no significant correlation between frequency of ICD coding and sensitivity of prescription registration (Figure 4).

On the X axis: Proportion of coded visits. On the Y axis: Proportion of patients with correct registration

On the X axis: Proportion of coded visits. On the Y axis: Number of patients with a correct combination of Prescription/ICD code registration respectively.

### Multilevel analysis for quantifying the relative importance of the different levels for the frequency of ICD-coding

Table 3 shows that the frequency of visits with ICD coding varied among the 24 HCCs from 42% to 93% with a median of 72%. The multilevel logistic regression (Table 4) showed that there was a high clustering of similar behaviour among physicians from the same HCC (MOR_HCC-PHYSICIAN _= 5.23). In other words, if a patient moved to a new physician working in a different HCC that had a higher propensity for ICD coding, the odds of registration will, in median, increase 5.23 times. Analysing the independent role of the physician and the HHC we observed that the larger component of variance was found at the physician level (MOR_-PHYSICIAN = _4.22; 95% CI 3.92-4.58).

**Table 3:** Characteristics of HCC, patient and visit level variables included in the multilevel analysis.

**Characteristics of HCCs**	Median	Range
*Visits, n*	13352	2842-33091
*Patients, n*	6350	1493-14573
*Physicians, n*	29	4-133
*Coding frequency, %*	72	42-93
		
**Characteristics of patients**	Numbers	Proportion
*Agegroup*		
-28	47357	31%
29-49	39129	25%
50-67	36357	24%
68-	31689	20%
*Sex*		
Female	83398	54%
Male	71105	46%
		
**Characteristics of visits**		
*ICD-coding*	Yes	No
*Visits, n(%)*		
All	245126 (70%)	103650 (30%)
Planned	94013 (65%)	49827 (35%)
Not Planned	151113 (74%)	53823 (26%)

Extracted from SPCD during 1^st ^May 2002 - 31^st ^October 2003

**Table 4:** Multilevel logistic regression analysis of frequency of ICD coded visits

	Model A	Model B
**Fixed effects**		OR (95% CI)
		
*Patient age group*		
1 (-28)	-	REF
2 (29-49)	-	0.86 (0.84-0.89)
3 (50-67)	-	0.84 (0.82-0.87)
4 (68-)	-	0.75 (0.73-0.77)
		
*Patient sex*		
Female	-	REF
Male	-	0.98 (0.96-1.00)
		
*Type of visit*		
Planned	-	REF
Not planned	-	1.44 (1.41-1.47)
		
**Random effects**		
		
HCC Variance (95% CI)	0.76 (0.40-1.54)	0.76 (0.41-1.50)
MOR (95% CI)	2.30 (1.82-3.26)	2.29 (1.84-3.22)
Physician Variance (95% CI)	2.28 (2.05-2.55)	2.25 (2.02-2.53)
MOR(95% CI)	4.22 (3.92-4.58)	4.19 (3.88-4.56)
HCC+Physician Variance	3.04	3.01
MOR	5.23	5.28
		
PCV		
HCC	-	0.3%
Physician	-	1.3%
HCC+Physician	-	0.9%
	303170.55	301079.69
DIC (MCMC)		

Outcome variable at the visit level, ICD coding (yes/no). Extracted from SPCD during 2002-2003 for the 24 Health Care Centres in Skaraborg Primary Care.

OR = odds ratio, CI = credible interval, MOR = median odds ratio, DIC = Deviance Information Criterion

Compared to planned visits, unplanned visits resulted more frequently in an ICD coding (OR 1.44; 95% CI 1.41-1.47). Moreover, compared to the youngest age group, older patients were less likely to get their visits ICD coded (OR 0.75; 95% CI 0.73-0.77).

The inclusion of individual characteristics at the physician level (model B) explained only a very small part of the higher level variance (PCV_HCC-PHYSICIAN _= 0.9%).

The DIC statistics showed that model B had a better model fit than model A.

## Discussion

The main findings of this study were that the sensitivity of ICD code registration varied between diagnoses, being highest for diabetes mellitus (89%) and hypertension (83%) and lowest for CHF (66%), and that there was a large variation between physicians and between HCCs in the frequency of ICD coding with the largest difference being between physicians.

The observed variation in sensitivity between different diagnoses is in line with previous studies [6,12]. A reason for the high sensitivity found for diabetes might be that diabetes has clearly defined and well known diagnostic criteria and is therefore more readily coded than other diagnoses with more complex diagnostic criteria, for which the physician may choose to record a note as free text but not select any specific ICD code. Thus, it is likely that nearly all diabetic patients attending an HCC can be identified in the database whereas identification of patients with CHF is more incomplete. Hypertension also had a high ICD coding frequency in the SPCD. The explanation for this finding could be that in Skaraborg primary care, a large project with a standardized protocol for screening and treatment of hypertension was inaugurated in the seventies [13,14], and most of the physicians therefore have a long tradition of diagnosing and managing hypertensive patients. Still the prevalence for hypertension was about half of what would be expected from earlier studies of the Skaraborg population aged 40-69 years [15]. Similarly, the prevalence of CHF was also half of that expected from the Treatment guidelines from the Swedish Medical Products Agency in 2006 [16] but on the same level as that reported in another study of computerized patient records in Swedish primary care [17]. Thus, when using databases such as the SPCD in quality assessments and research, several aspects may need to be considered, including local routines and initiatives that may increase the registration of certain diagnoses.

Even though there are several possible sources of error in the prescriptions register, such as failure to register when medication is terminated, the overall quality of the information on prescriptions seems to be better than for the ICD coding. This is probably due to the fact that medication is automatically registered when the prescription is printed. There was an 88% mean registration sensitivity for prescriptions, even for rather complex medications. This value was similar to that found for diabetes ICD coding.

The frequency of coded visits is the most frequently used quality measure for ICD coding and theoretically it should be correlated to the coding of specific diagnoses. This was true for some of the diagnoses (hypertension and CHF) but not for others. This could be explained by different prerequisites for ICD coding. Sensitivity of hypertension and congestive heart failure coding showed a weak correlation to the overall coding frequency, but because the number of observations of CHF was low these figures should be interpreted with care. Diabetes mellitus had high coding frequencies in all HCCs, but there was no correlation with the overall coding frequency. Thus, the coding frequency of patient visits is not always a useful measure of completeness of ICD code registration in chronic diseases. Different types of diagnoses need to be validated separately.

The frequency of ICD coding seemed to depend largely on physicians and HCCs, with the greatest part of variability found at the physician level. Part of this variation could in fact be at the patient level since a patient can have several visits, however, as the residuals at the patient level were not normally distributed we excluded this level from the analysis. A complementary analysis using Generalized Estimation Equations and Alternating Logistic Regression [18] also showed that the clustering at the patient level was small (pair wise odds ratio of 1.15) and that the exclusion of the patient level will have only limited effect on the variance at the higher levels.

Our results suggest that physicians may be a more effective target than HCCs for interventions intended to improve ICD code registration.

Even though the inclusion of individual characteristics such as age and type of visit were conclusively associated with ICD coded visit, it did not explain any part of the variance at the higher levels. The lower coding rates in planned visits might be explained by the greater complexity of the medical problems addressed during the planned visits in comparison with the unplanned visits. In the same way lower coding rates among the elderly could be attributed to their more complicated and time consuming medical conditions.

The result of a validation study is usually expressed by sensitivity, specificity and positive predictive value (PPV) [6,19]. When coding in the PDIII patient records, the assigned ICD codes are stored in the diagnosis register of the medical record database. However, at the selection of an ICD code, the software automatically records a notation in the text section, which was used as gold standard in this study. Therefore we found it inappropriate to distinguish between true (A) and false (B) positive cases as the former, per definition, amounts to 100% (Figure 2). This could be overcome by discarding the automated text notations from the review but in this study we chose to include everything. With this approach it was not possible to calculate specificity and PPV. Since the assigned codes reflect the opinion of the physician, a more thorough and objective validation of the quality of coding would have to include comparison of the medical outcomes of the individual patients with the diagnostic criteria for the relevant diagnoses. This was not done in this study, and therefore we can only reflect on the registration performance of the physicians, but not their diagnostic capabilities.

The observed difference in ICD coding on the higher levels could be accounted for by differences in staffing of the HCCs. Lack of physicians and many locum doctors might influence time spent on ICD code registration. This is a very important issue to address in further studies as in Sweden the shortage of general practitioners will probably remain. It is ultimately the individual physician who is responsible for selecting and entering an ICD code, and since there are no external incentives for coding we expected to find a variation in coding practice among physicians. This was also demonstrated in the study. A further analysis including factors such as the physicians' age, training and years in the profession as well as interviews addressing their attitudes towards ICD code registration and perception of workload could further clarify the reasons for the variation. The lack of external incentives for coding during this time period give us no reason to believe that other than purely medical considerations would affect the coding. This fact minimizes the risk of coding bias due to economical considerations when coding, but also results in low coding rates. In 2009, such incentives were introduced by making HCC reimbursement to a large extent dependent on ICD coding, using the ACG (Adjusted Clinical Group) system [20]. While this will probably increase coding sensitivity and reduce variation, it may also jeopardize the correctness of coding. In a future study we aim to investigate what impact this new reimbursement system will have on coding frequencies and coding patterns.

## Conclusions

The frequency of ICD code registration varies between physicians and health care centres, but also between different diagnoses. Validation of ICD codes is necessary for each specific diagnosis. In the present study, diabetes was most frequently registered while congestive heart failure was least frequently registered. The frequency of ICD coding seemed to depend largely on physicians and HCCs, with the greatest part of variability found at the physician level. Increased frequency and quality of ICD code registration is important for future quality assessments and might be achieved by interventions directed towards the physicians.

## Competing interests

The authors declare that they have no competing interests.

## Authors' contributions

PH, UL and JM participated in the design of the study. PH performed the validation of the medical records, and PH and HO were responsible for the statistical analyses. All authors participated in interpretation of the data. PH, KBB and JM drafted the manuscript. All authors critically reviewed and gave final approval to the manuscript.

## Pre-publication history

The pre-publication history for this paper can be accessed here:

http://www.biomedcentral.com/1472-6947/10/23/prepub
